# The impact of Covid-19 on malaria services in three high endemic districts in Rwanda: a mixed-method study

**DOI:** 10.1186/s12936-022-04071-3

**Published:** 2022-02-14

**Authors:** Dieudonne Hakizimana, Christian Ntizimira, Aimable Mbituyumuremyi, Emmanuel Hakizimana, Hani Mahmoud, Pascal Birindabagabo, Clarisse Musanabaganwa, Diane Gashumba

**Affiliations:** 1 University of Global Health Equity (UGHE), Kigali, Rwanda; 2 Health Concepts and Innovation Solutions (HCIS), Kigali, Rwanda; 3 Rwanda Biomedical Center (RBC), Kigali, Rwanda; 4 Resolve To Save Lives, New York, NY USA

**Keywords:** Malaria, Covid-19, Service delivery, Mixed-methods, Rwanda

## Abstract

**Background:**

Rwanda has achieved impressive reductions in malaria morbidity and mortality over the past two decades. However, the disruption of essential services due to the current Covid-19 pandemic can lead to a reversal of these gains in malaria control unless targeted, evidence-based interventions are implemented to mitigate the impact of the pandemic. The extent to which malaria services have been disrupted has not been fully characterized. This study was conducted to assess the impact of Covid-19 on malaria services in Rwanda.

**Methods:**

A mixed-methods study was conducted in three purposively selected districts in Rwanda. The quantitative data included malaria aggregated data reported at the health facility level and the community level. The data included the number of malaria tests, uncomplicated malaria cases, severe malaria cases, and malaria deaths. The qualitative data were collected using focus group discussions with community members and community health workers, as well as in-depth interviews with health care providers and staff working in the malaria programme. Interrupted time series analysis was conducted to compare changes in malaria presentations between the pre-Covid-19 period (January 2019 to February 2020) and Covid-19 period (from March 2020 to November 2020). The constant comparative method was used in qualitative thematic analysis.

**Results:**

Compared to the pre-Covid-19 period, there was a monthly reduction in patients tested in health facilities of 4.32 per 1000 population and a monthly increase in patients tested in the community of 2.38 per 1000 population during the Covid-19 period. There was no change in the overall presentation rate for uncomplicated malaria. The was a monthly reduction in the proportion of severe malaria of 5.47 per 100,000 malaria cases. Additionally, although healthcare providers continued to provide malaria services, they were fearful that this would expose them and their families to Covid-19. Covid-19 mitigation measures limited the availability of transportation options for the community to seek care in health facilities and delayed the implementation of some key malaria interventions. The focus on Covid-19-related communication also reduced the amount of health information for other diseases provided to community members.

**Conclusion:**

The Covid-19 pandemic resulted in patients increasingly seeking care in the community and poses challenges to maintaining delivery of malaria services in Rwanda. Interventions to mitigate these challenges should focus on strengthening programming for the community and home-based care models and integrating malaria messages into Covid-19-related communication. Additionally, implementation of the interrupted interventions should be timed and overlap with the malaria transmission season to mitigate Covid-19 consequences on malaria.

## Background

Although malaria continues to be a significant global public health issue affecting around 229 million people each year [1], there have recently been remarkable achievements in reducing its morbidity and mortality in most countries including Rwanda. From 2005 to 2012, malaria control efforts in Rwanda achieved a significant reduction in malaria incidence from 170 cases per 1000 population at risk in 2005 to 48 per 1000 population at risk in 2012 (from 1.5 million cases in 2005 to 567,407 in 2012). Despite these efforts, Rwanda has experienced a dramatic upsurge in malaria cases between 2012 and 2017. During that period, malaria cases increased from 48 per 1000 population in 2012 to 406 per 1000 population at risk in 2017 (from 567,407 in 2012 to 4.8 million in 2017 [2–4].

The hypothesized factors of that increase include general malaria increase in the eastern African region, government expansion of rice cultivation in marshlands, inconsistency in vector control methods, mosquito resistances, low insecticide-treated nets (ITN) coverage, a general increase in the number of patients consulting health facilities due to improved access to health insurance and improved availability of malaria commodities (rapid diagnostic tests and artemisinin-based combination treatment) [2, 4].

The Rwanda malaria control programme developed a contingency plan to curb that increase. Strategies include enabling community health workers to treat all people in the community, free malaria diagnostics and treatment for low-income populations, mass ITN distribution, expansion of indoor residual spraying (IRS) from three to five districts and later to 12 districts, and use of organophosphate insecticide instead of carbamate, and engagement with other sectors including the Ministry of Agriculture to mitigate the effects of farming practices and environmental changes [2]. These improved malaria control strategies have helped to curb the trend in malaria cases with a reduction from 406 per 1000 population at risk in 2017 to 145 per 1000 population at risk in 2020 (from 4.8 million malaria cases in 2017 to 1.8 million in 2020). This led to a remarkable reduction (around 60%) in both severe malaria cases and malaria-related deaths in the last three years. The country has set a goal to reduce malaria mortality and morbidity by at least 50% of the 2019 levels by 2024 [5, 6].

Rwanda confirmed its first Covid-19 case on March 12, 2020. Immediately, the government instituted Covid-19 response measures including contact tracing, quarantine, and countrywide lockdown from March 21, 2020, to May 04, 2020, and other subsequent and targeted lockdowns. Only essential services were allowed to operate such as markets and health facilities [7–13]. As of November 2021, nearly 100,000 people were infected with Covid-19 and over 1,300 people died of Covid-19 in Rwanda [14].

The Covid-19 pandemic has likely impacted malaria service delivery in many settings for several reasons. The efforts to curb the pandemic draw from existing limited financial resources, leaving other services underfunded. Moreover, responding to pandemics requires an additional health workforce that was already scarce. Due to limited personal protective equipment (PPE) and testing capacity in some settings, the available health providers are also likely to be exposed and/or infected. Subsequent isolation, quarantine, or sometimes death further exacerbates the issue of health provider scarcity. Additionally, Covid-19 mitigation measures such as lockdown in countries may affect not only the international and local supply chain—including commodities manufacturing, procurement, shipping, and distribution—but also may affect malaria services utilization. For instance, patients may not seek malaria care due to limited transportation options or fear of contracting Covid-19. Patients may also seek care too late, when severely ill, and thus at increased risk of mortality. Recent studies estimated that the number of deaths from malaria could double if the world’s attention continues to be strictly on Covid-19 [15, 16].

Existing evidence suggests that during health crises, unless targeted efforts are put in place, health services may not recover to pre-health crisis levels [17]. An enhanced understanding of the effects of the Covid-19 pandemic on health services is vital to better design evidence-based interventions and mitigate its negative impacts. This study was conducted to assess the impact of Covid-19 on malaria services to inform the design of interventions to mitigate the pandemic’s consequences on malaria services while continuing to support effective response to Covid-19.

## Methods

### Study setting

Rwanda is an East African country with an estimated population of 10.5 million in 2012, projected to be around 12.5 million in 2019 [18, 19]. The country is subdivided into 5 provinces (North, East, West and South, and Kigali City). Each province is subdivided into districts and there are 30 districts in total, in which malaria is highly endemic in 12 districts. The Rwandan health system is structured in a traditional hierarchical cascade as follows: community > health centre > district hospital > referral and specialized hospitals. Malaria interventions in Rwanda include core interventions such as (1) mosquito net distributions through annual campaigns and in routine services such as antenatal care and Expanded Programme of Immunization, (2) IRS in high burden districts, and (3) expanded access to timely diagnostic and treatment services at health facilities and at the community level, known as Home Based Management of Malaria. Supplemental interventions include behaviour change communication to improve knowledge and awareness of community members, larviciding in some targeted areas using drones, and use of mosquito repellent products [2].

**Fig. 1:**
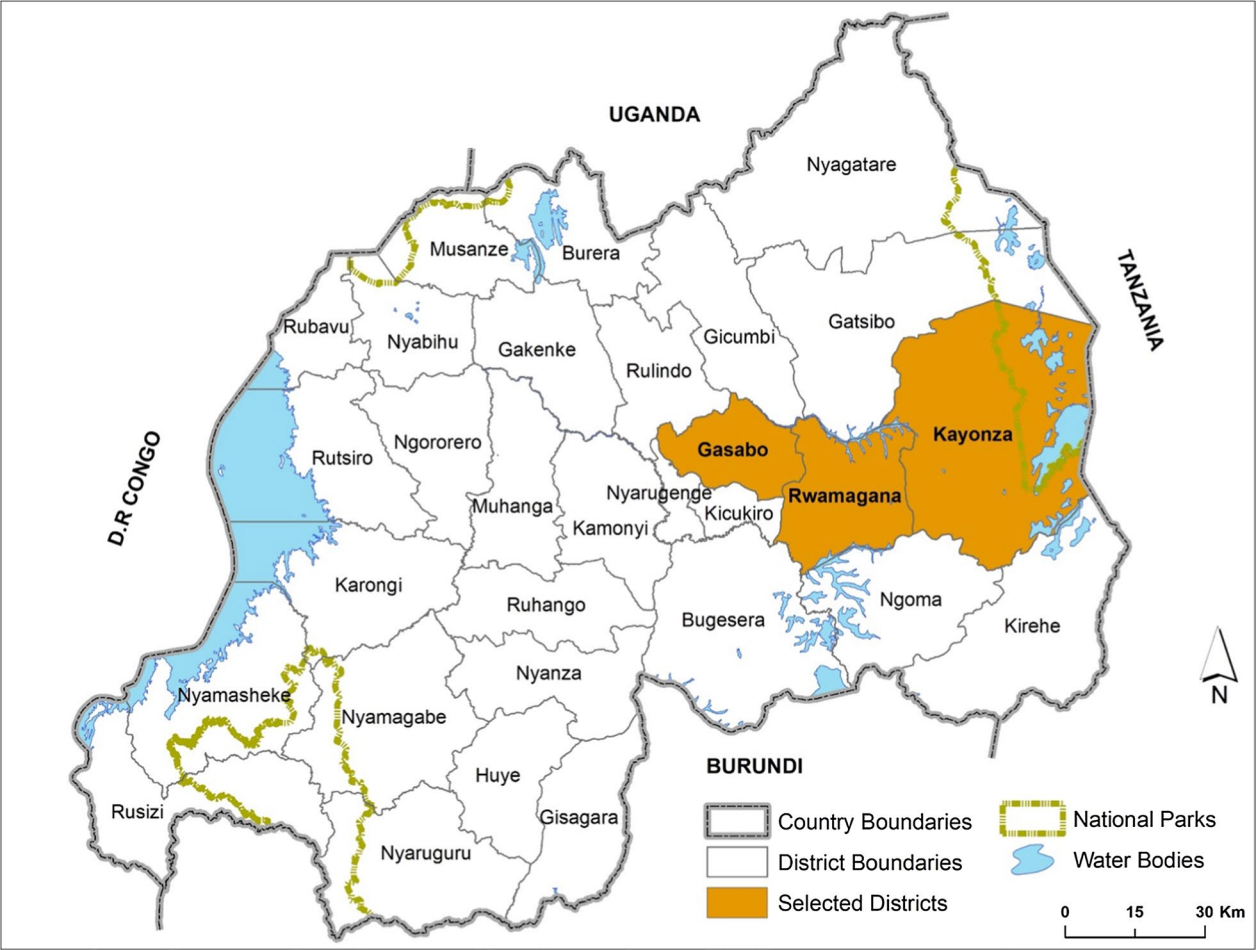
Rwanda map highlighting study districts. (This Map of Rwanda was created by the study team using Rwanda shapefile and ArcGIS Pro version 2.5) [46, 47]

The work presented here is part of a larger project to assess and mitigate the effects of the Covid-19 pandemic on Rwanda’s health system. The project was implemented from August to December 2020 in three districts: Rwamagana and Kayonza in Eastern Province, and Gasabo in Kigali city (Fig. 1). The Rwanda Biomedical Center selected these districts purposively, as part of its framework for the management of implementation partners, which allocates to each partner a specific geographic area of implementation for efficiency and to avoid duplication of activities between partners. The size of the population varies between the districts. The estimated population in 2020 was 637,686 in Gasabo district, 414,427 and 377,463 in Kayonza and Rwamagana district respectively [20]. The three districts are semi-urban. Although Gasabo district is among the district of the capital city, only 16% is a developed urban area, leaving 84% of its part as rural [21]. Subsistence agriculture is the main economic activity in these districts, with businesses in urban developed areas in each district. According to Rwanda integrated household living conditions survey in 2017, 15.8% of the Gasabo population were living in poverty, 18.9% in Rwamagana, and 26.7% in Kayonza; the national average being 36.7% [22]. Malaria is highly endemic in these three districts. According to data reported in the Rwanda malaria control programme 2020 report (unpublished data), there were 122,103 malaria reported cases in Gasabo district, or 191 cases per 1000 population at risk. In Kayonza district, 162,405 malaria cases were reported in 2020 and 93,042 malaria cases were reported in Rwamagana district, equivalent to 392 cases and 247 cases per 1000 population at risk in Kayonza district and in Rwamagana district, respectively. Similar malaria interventions are implemented in each district. However, the two districts of the Eastern province (Rwamagana and Kayonza) receive indoor residual spraying as part of malaria prevention while the Gasabo district in Kigali city receives a new generation of mosquito nets, the effect of which is believed to be comparable to indoor residual spraying [23]. The public health facilities available in these districts are one district hospital and 15 health centers in the Rwamagana district, two district hospitals and 14 health centers in Kayonza district, and one district hospital, one referral hospital, one specialized hospital, and 15 health centers in the Gasabo district. These districts are representative of other malaria high endemic districts in Rwanda. They are less representative of medium and low malaria-endemic districts.

### Study design

In this mixed-methods study, the quantitative component assessed changes in usage of malaria services, comparing the malaria presentations in the pre-Covid-19 period and the malaria presentations after the first Covid-19 case was diagnosed in Rwanda in March 2020. Fourteen months of data (January 2019 to February 2020) was used as the pre-Covid-19 period and nine months of data as the Covid-19 period (from March 2020 to November 2020). This is because, during that Covid -19 period, strict public health and social measures to mitigate Covid-19 were implemented across the districts whereas, after November 2020, the government started varying measures according to the epidemiological situation in each district [7–13].

The qualitative component explored the barriers community members faced in accessing malaria services during the period of lockdown, as well as the perspectives of Health Care Providers (HCPs), Community Health Workers (CHWs), and community members on health system readiness to ensure minimal interruption of the implementation of malaria interventions despite the lockdown.

### Data sources and data collection procedures

Both primary and secondary data were used in this study. The quantitative secondary data were aggregated data on malaria treatment reported in the Health Management Information System (HMIS) and the Système Informatique de Santé Communautaire (SIS-COM). Both systems are electronic nationwide databases used to report service delivery in Rwanda monthly. HMIS is used for reporting health facility data while SIS-COM is used to report CHW-related activities. The quality of data reported in both systems is high [24]. The malaria data included the number of malaria tests conducted at health facilities and in the community separately, the number of uncomplicated malaria cases diagnosed in health facilities and the community, and the number of severe malaria cases and malaria deaths.

Qualitative data were collected during August–September 2020, conducting focus group discussions with community members and CHWs, as well as in-depth interviews with key informants that included HCPs and staff working in the malaria programme at the central level. The key informant interview participants were selected purposively based on their involvement in malaria service provision. They included personnel working in the malaria programme at the central level and district level, those working with CHWs at the district level, health facilities managers, and in charge of CHWs at the community level in selected health centres. For the focus group discussions, the CHWs and community members were selected by the health facilities managers according to their convenience to participate in the focus group discussions. The health facility managers were requested to diversify participants and include both those living near the health facility and those living far from the health facility, to capture all experiences.

The semi-structured interviews and focus group discussion guides used in data collection covered key themes including whether the health system was sufficiently prepared to ensure continuous malaria activities during the period of Covid-19 mitigation measures, and the barriers to access malaria services by the community members during the period of lockdown. The data collectors who were fluent in both English and Kinyarwanda (the local language) were used for data collection. They received a 2-day training on data collection tools before the data collection. Both in-depth interviews and focus groups were conducted in the local language and were audio-recorded.

### Data management and analysis

The data from HMIS, and SIS-COM systems were extracted and exported them into Stata version 14.2 (StataCorp, College Station, Texas) for cleaning and to calculate malaria indicators. To measure the effect of Covid-19 on malaria service use, interrupted time series analysis was conducted, which is among quasi-experimental study designs with advantages of using pre-existing data, and account for previous trends in the outcomes, which are very frequent in malaria data [25–27].

For uncomplicated malaria, the rate per 1000 population was calculated by dividing the number of patients reported per month by the estimated total population for the three districts. The district population was estimated using the medium population growth rates based on the 2012 census data as reported by the National Institute of Statistics of Rwanda [18]. The population denominators were used for each year separately. The combined population for all districts was 1,397,038 and 1,429,640 for the year 2019 and year 2020, respectively. For severe malaria, the rate per 100,000 malaria cases was calculated by dividing the number of patients with severe malaria by the total malaria cases reported per month. Moreover, the presentation rate for testing (suspected cases) per 1,000 population in the health facilities and the community were calculated separately by dividing the number of patients tested in the health facilities and the community by the projected population. The proportion of suspected malaria cases that received a parasitological test at the community level was calculated by dividing the number of tests performed at the community level by the total malaria tests. To calculate the proportion of malaria cases diagnosed at the community level, the positive cases at the community level were divided by the total positive cases. The overall malaria test positivity rate and the malaria test positivity rate at the community level and at the health facility level separately were calculated by dividing the total positive tests by the total tests performed. To calculate the case fatality rate, the number of deaths were divided by the total number of malaria cases. For each indicator, interrupted time series analysis was conducted by fitting generalized least squares models to compare trends and levels between the pre-Covid-19 period and the Covid-19 period, considering autocorrelation lag. Autocorrelation was assessed by plotting autocorrelation and partial autocorrelation functions. The time-series analysis was conducted using R 4.0.2, the “nlme” and “car” packages [28, 29]

The thematic analysis included 12 focus group discussions (six conducted with CHWs, six with community members) and 22 interviews (20 conducted with HCPs and staff working in malaria services at the district level, and two with staff from the malaria programme at the central level). The constant comparative method was used for thematic analysis of the qualitative data [30, 31]. Audio recordings were transcribed in Kinyarwanda. The research team members fluent in both English and Kinyarwanda first read 10 transcripts and inductively developed a codebook. The codebook was used to highlight key excerpts from the transcripts and modified it as new themes emerged from the data during the analysis process. The key themes were reported and supplemented them with quotes from participants. The quotes used in reporting were translated into English by a professional translator and then verified by the principal investigators for accuracy. Qualitative analysis was conducted using Dedoose Version 4.12. This study was reviewed and approved by the Rwanda National Ethics Committee, and participants consented prior to their involvement in the study.

## Results

### Time-series analysis of malaria services utilization, before and during Covid-19

**Fig. 2:**
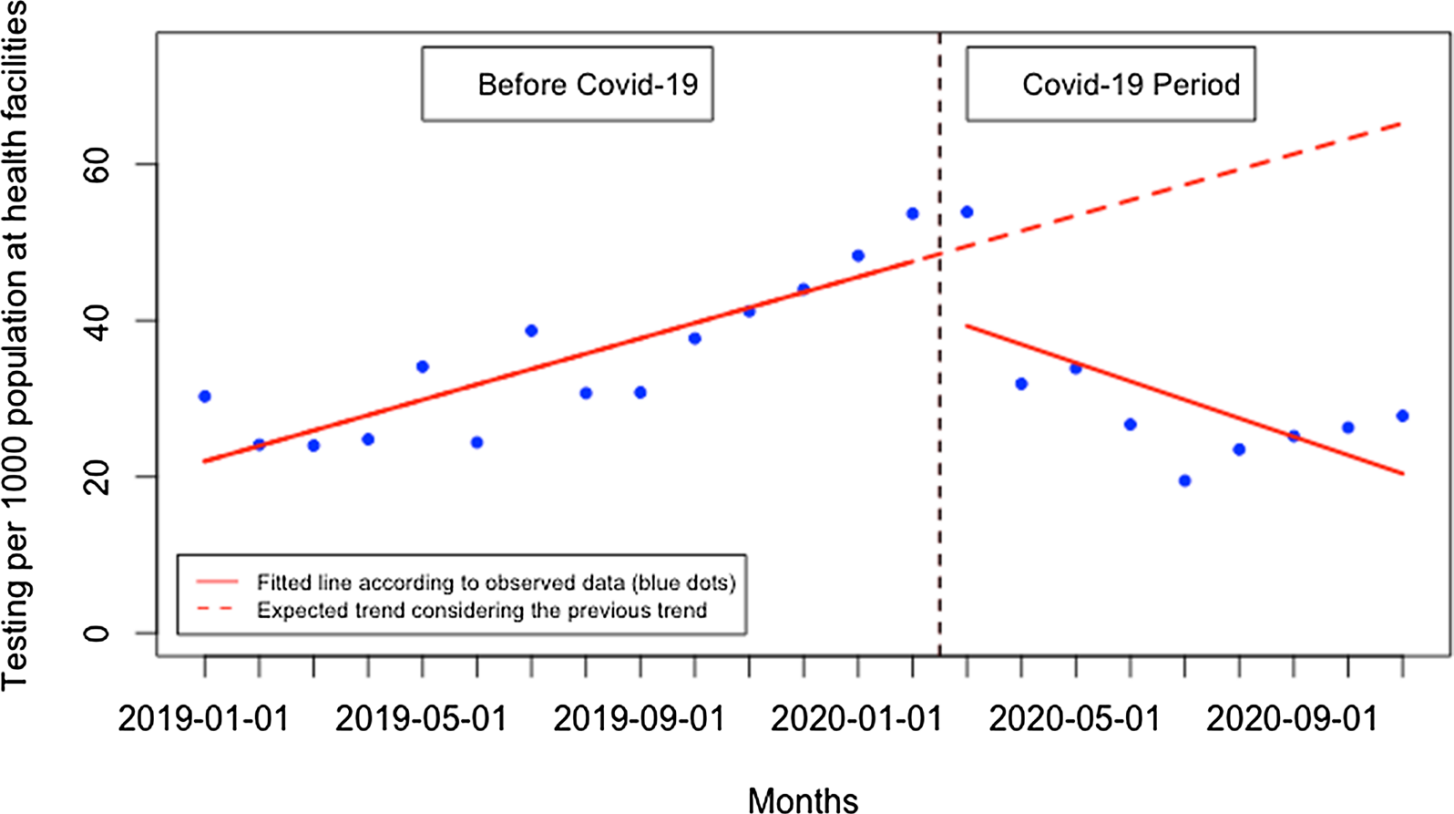
Presentation rate for malaria testing (per 1000 population) at the health facility level

**Fig. 3:**
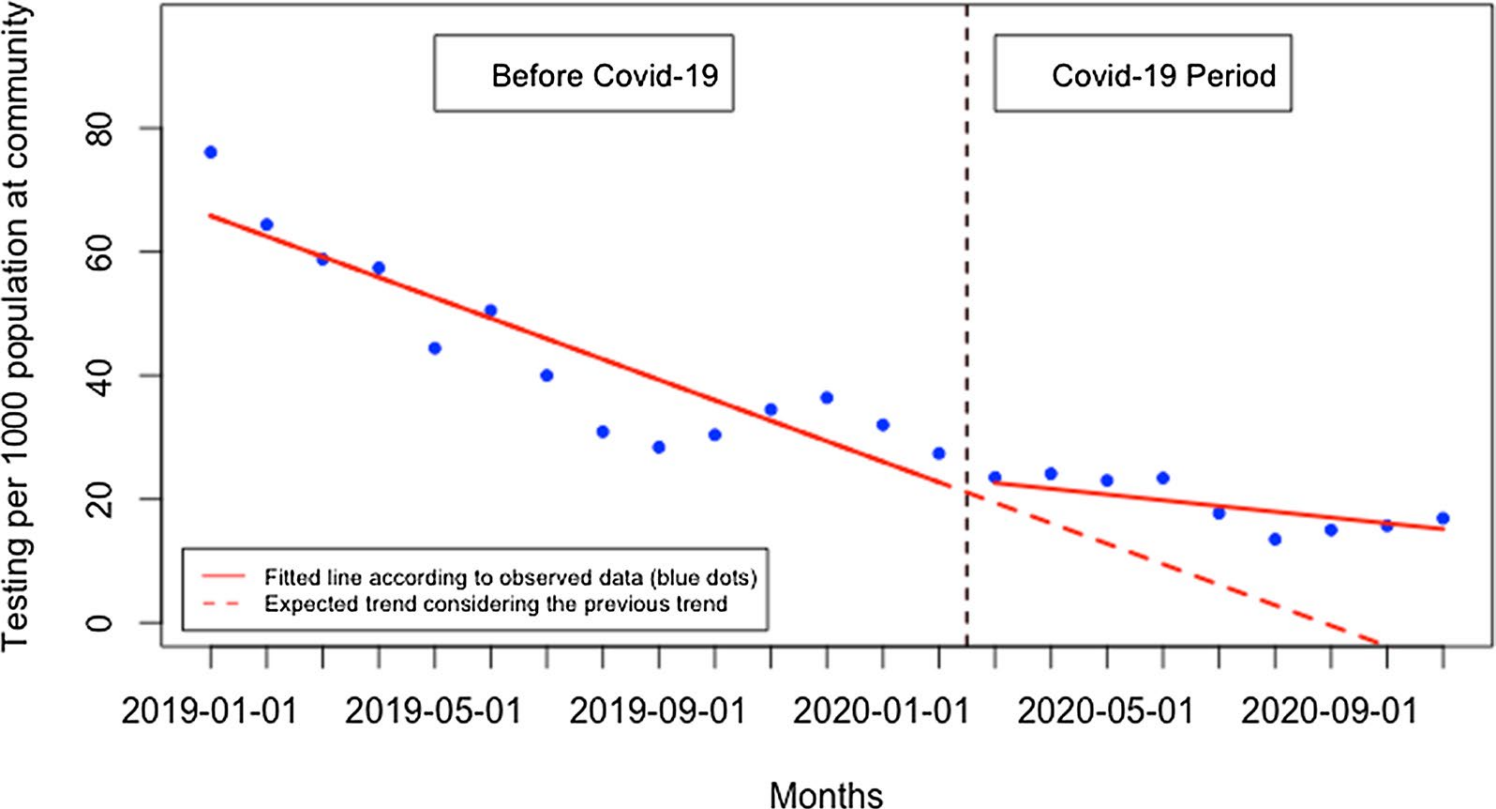
Presentation rate for malaria testing (per 1000 population) at the community level

**Fig. 4:**
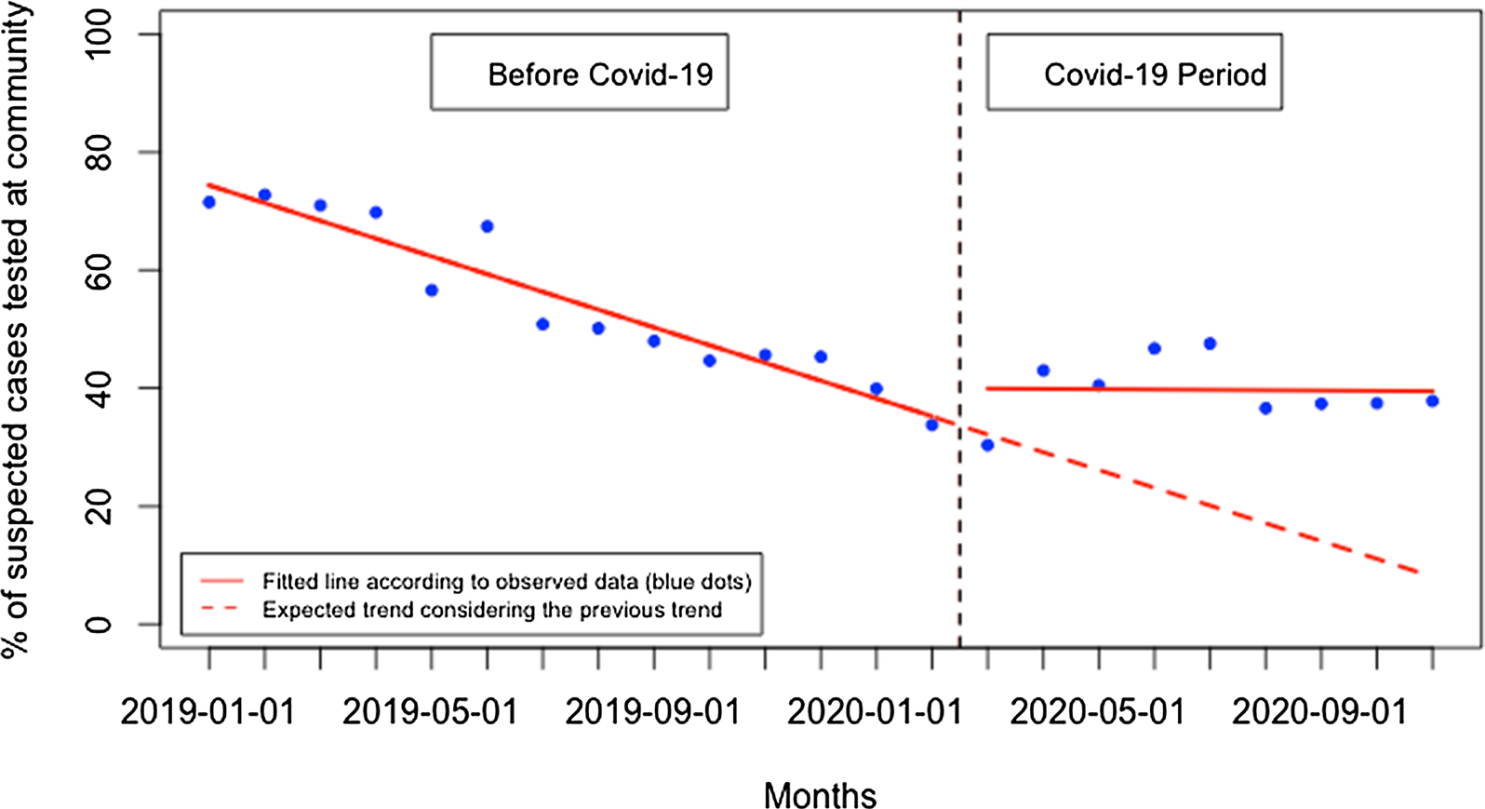
Proportion of suspected malaria cases that received a parasitological test at the community level

**Fig. 5:**
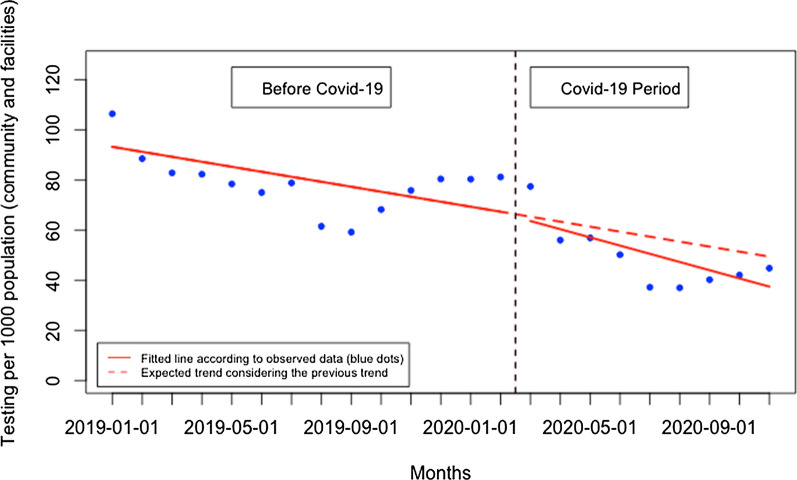
Presentation rate for malaria testing (per 1000 population)—community and health facilities combined

During the post-Covid-19 period compared to the pre-Covid-19 period, there was a significant reduction in patients tested in health facilities and an increase in patients tested in the community. Although there was no significant change in the presentation rate of patients for malaria testing at the health facility level immediately when Covid-19 was first reported in Rwanda, there was subsequently a constant monthly significant reduction in the presentation rate of patients for malaria testing of 4.32 per 1000 population (95% CI − 6.25, − 2.40, p: < 0.001) (Fig. 2). At the community level, there was also no significant change immediately when Covid-19 was reported in Rwanda, but there was a constant significant increase in the presentation rate of patients for malaria testing at the community level of 2.38 per 1000 population during the Covid-19 period, (95% CI 0.34, 4.41, p: 0.033) (Fig. 3). This led to a significant increase in the proportion of suspected malaria cases that received a parasitological test at the community level of 2.96% during the Covid-19 period (95% CI 1.49, 4.42, p: < 0.001) (Fig. 4). When both health facilities and community tests were combined (Fig. 5), there was no significant change in the presentation rate of patients for malaria testing immediately when Covid-19 was first reported in Rwanda (− 0.43, 95% CI − 18.29, 17.42, p: 0.962) as well as during the covid period (− 1.28, 95% CI − 5.76, 3.20, p: 0.582).

**Fig. 6:**
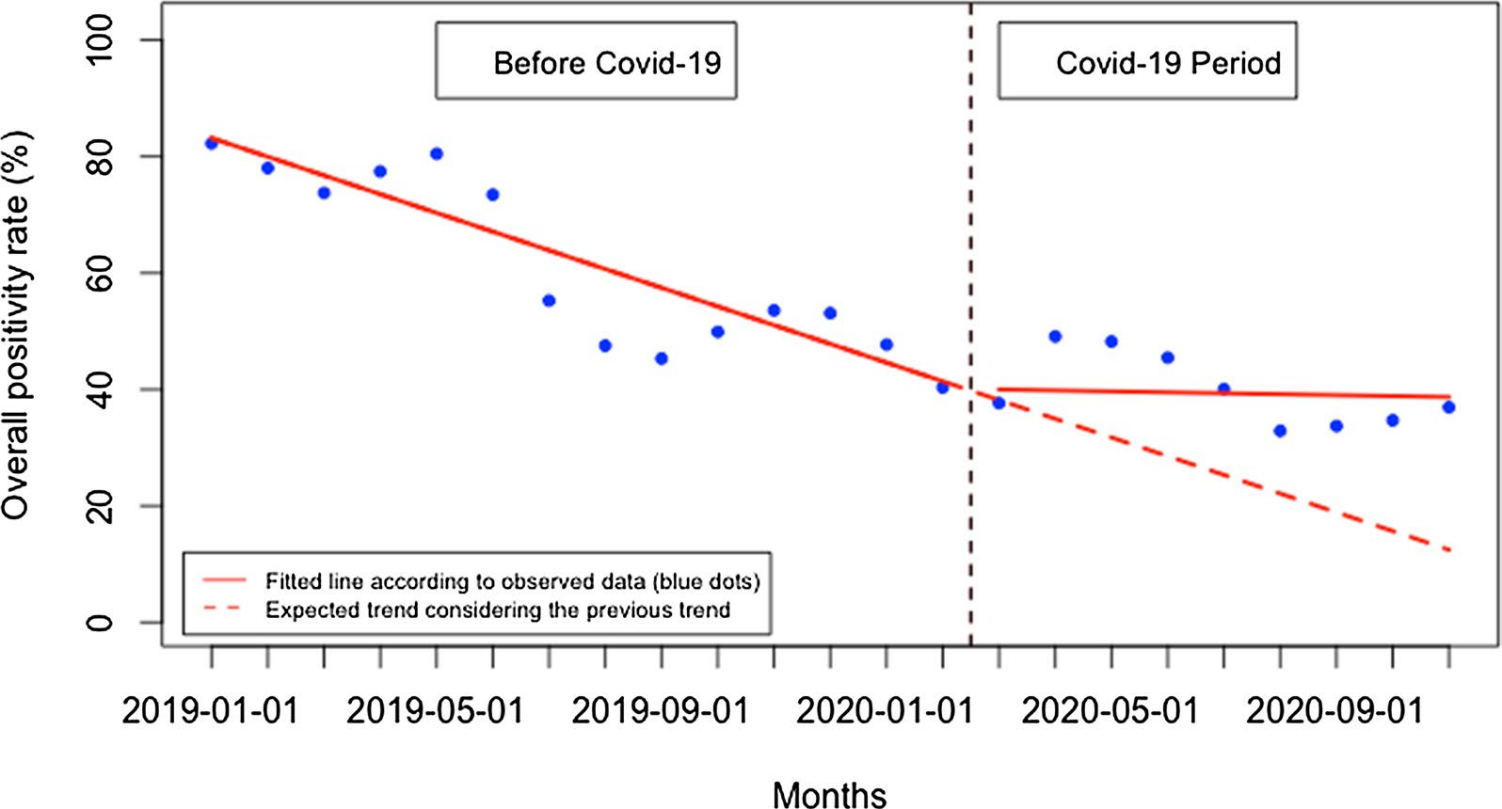
Overall malaria test positivity rate (%)

**Fig. 7:**
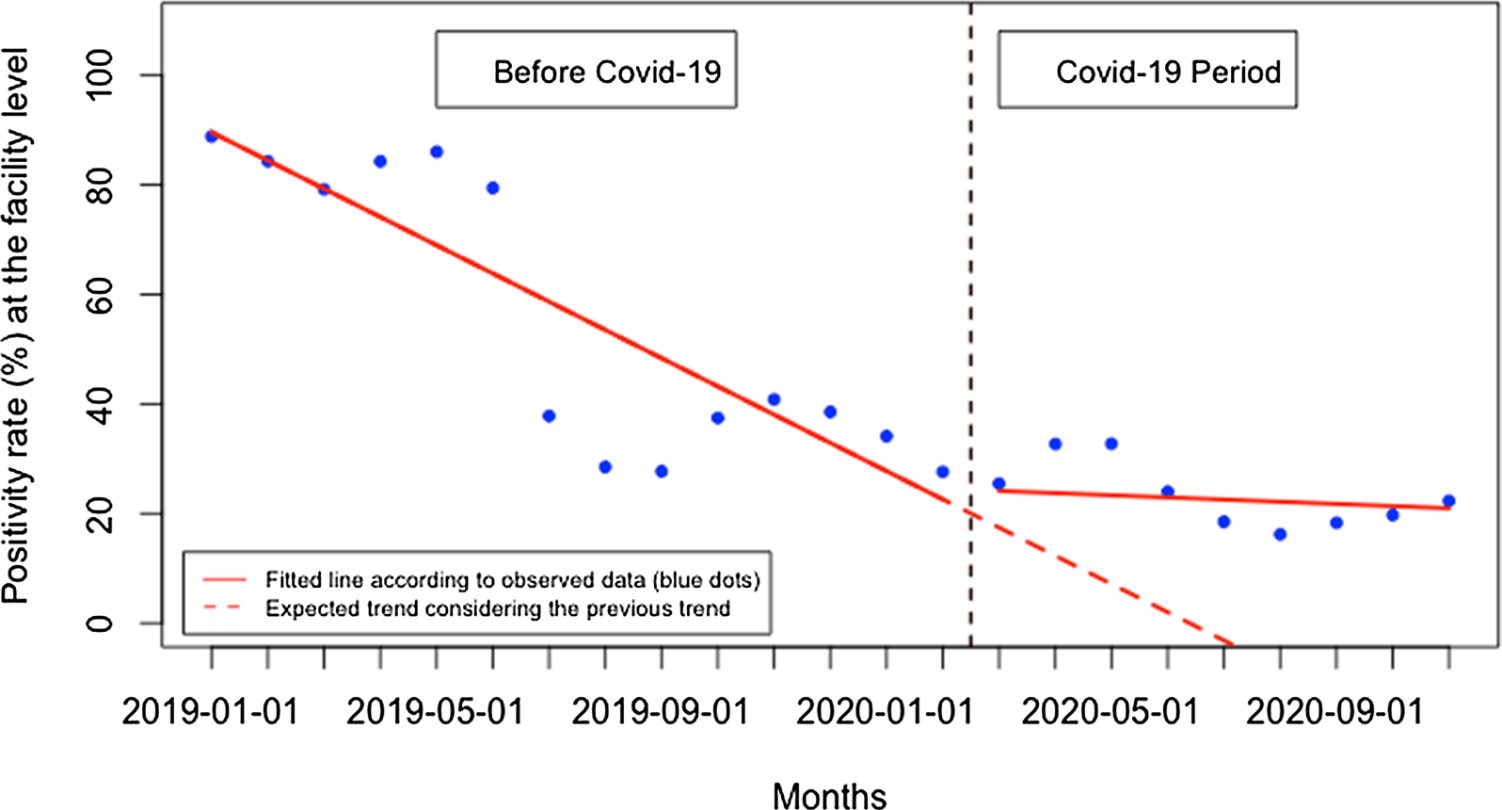
Malaria test positivity rate (%) at the health facility level

**Fig. 8:**
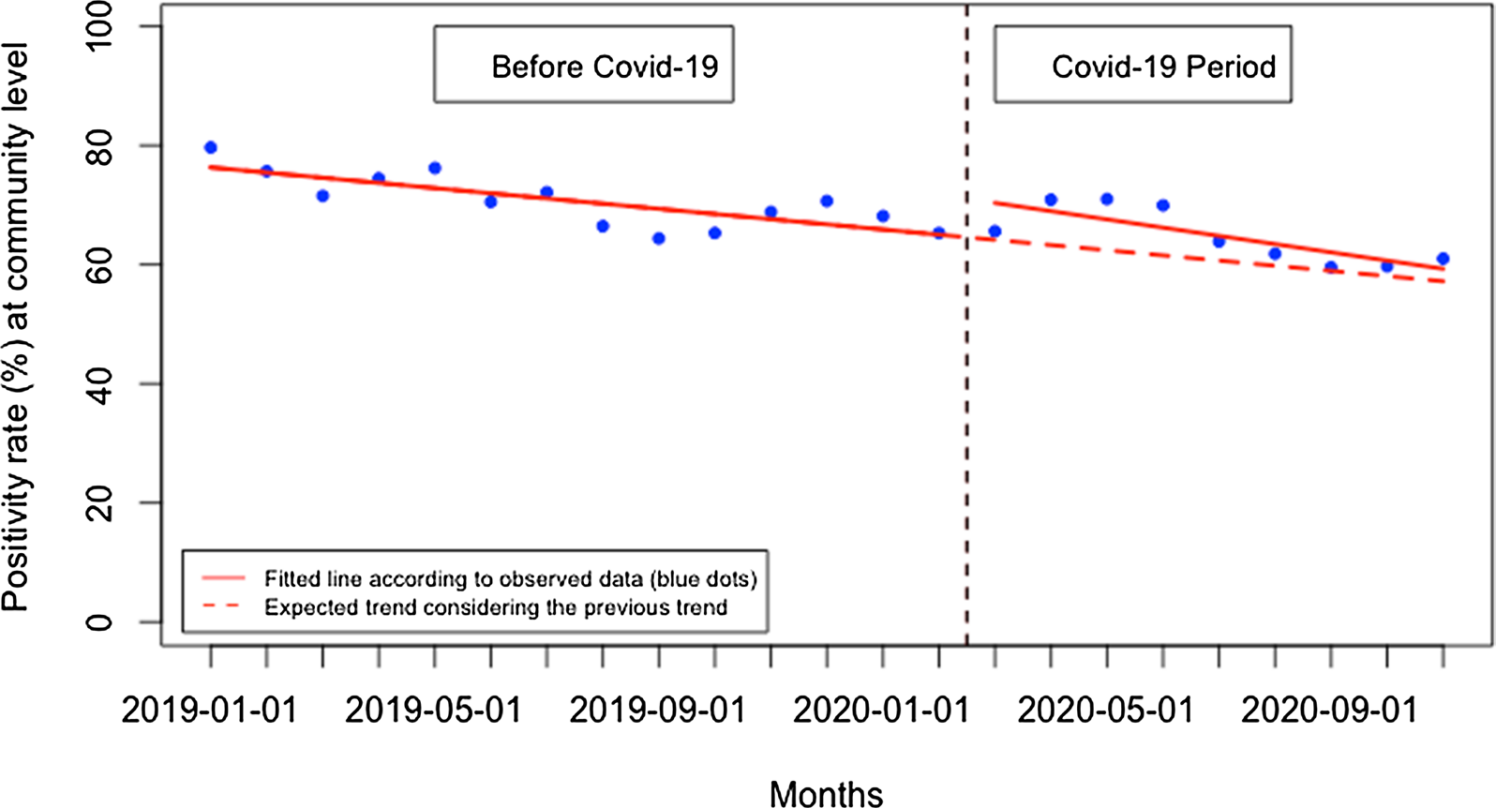
Malaria test positivity rate (%) at the community level

The overall malaria test positivity rate did not significantly change immediately when Covid-19 was first reported in Rwanda, but there was a significant monthly increase during the Covid-19 period of 3.05% (95% CI 1.22, 4.88, p: 0.004) (Fig. 6). When disaggregated, at the health facility level (Fig. 7), malaria test positivity rate did not significantly change immediately when Covid-19 was first reported in Rwanda, but there was a significant monthly increase during the Covid-19 period of 4.75% (95% CI 1.36, 8.15, p: 0.012). Contrarily, at the community level (Fig. 8), the malaria test positivity rate significantly increased to 6.72% immediately when Covid-19 was first reported in Rwanda (95% CI 1.13, 12.30, p: 0.020) but there was no significant monthly change during the Covid-19 period (− 0.51, 95% CI − 1.42, 0.40, p: 0.253).

**Fig. 9:**
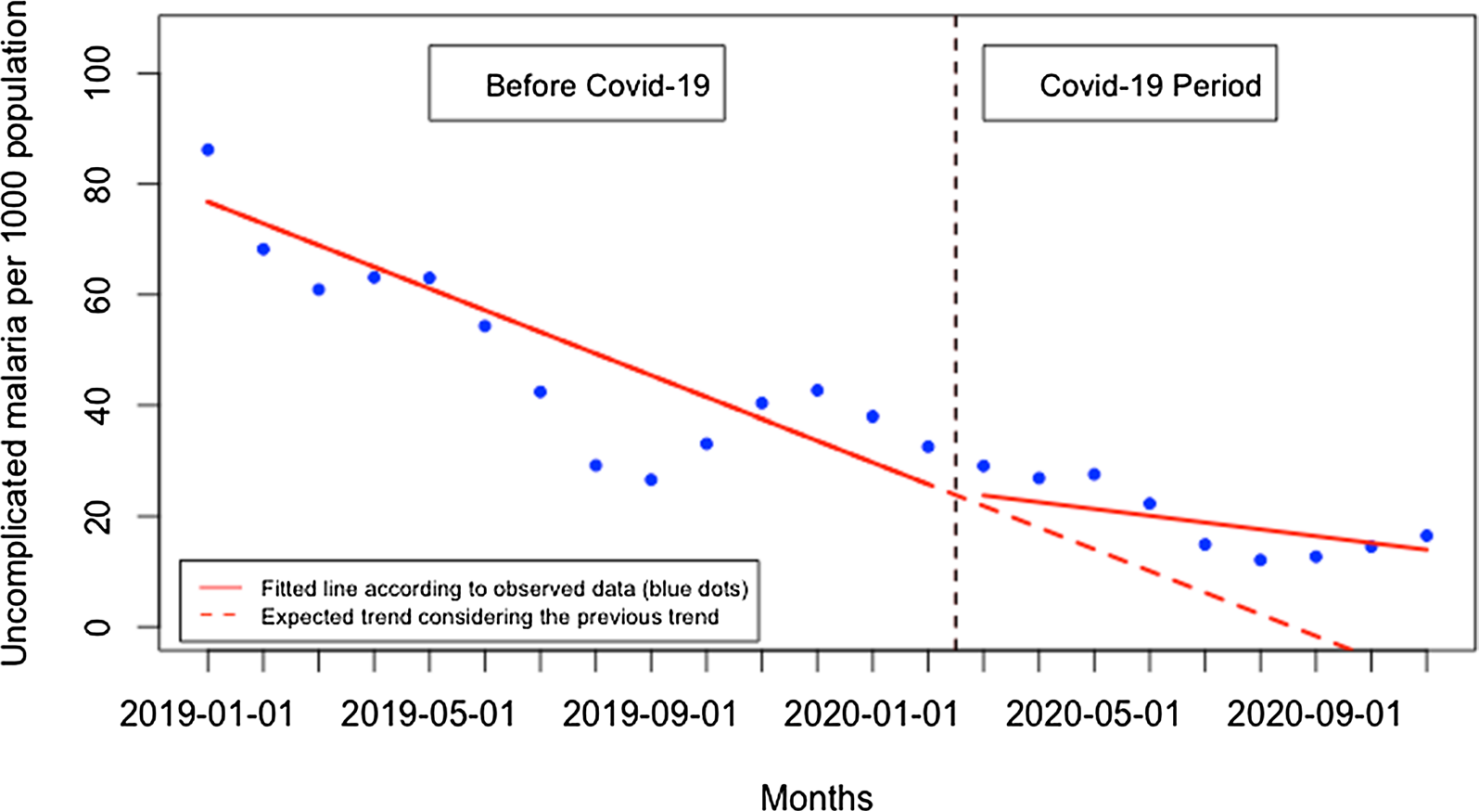
Presentation rate for uncomplicated malaria cases (per 1000 population)

**Fig. 10:**
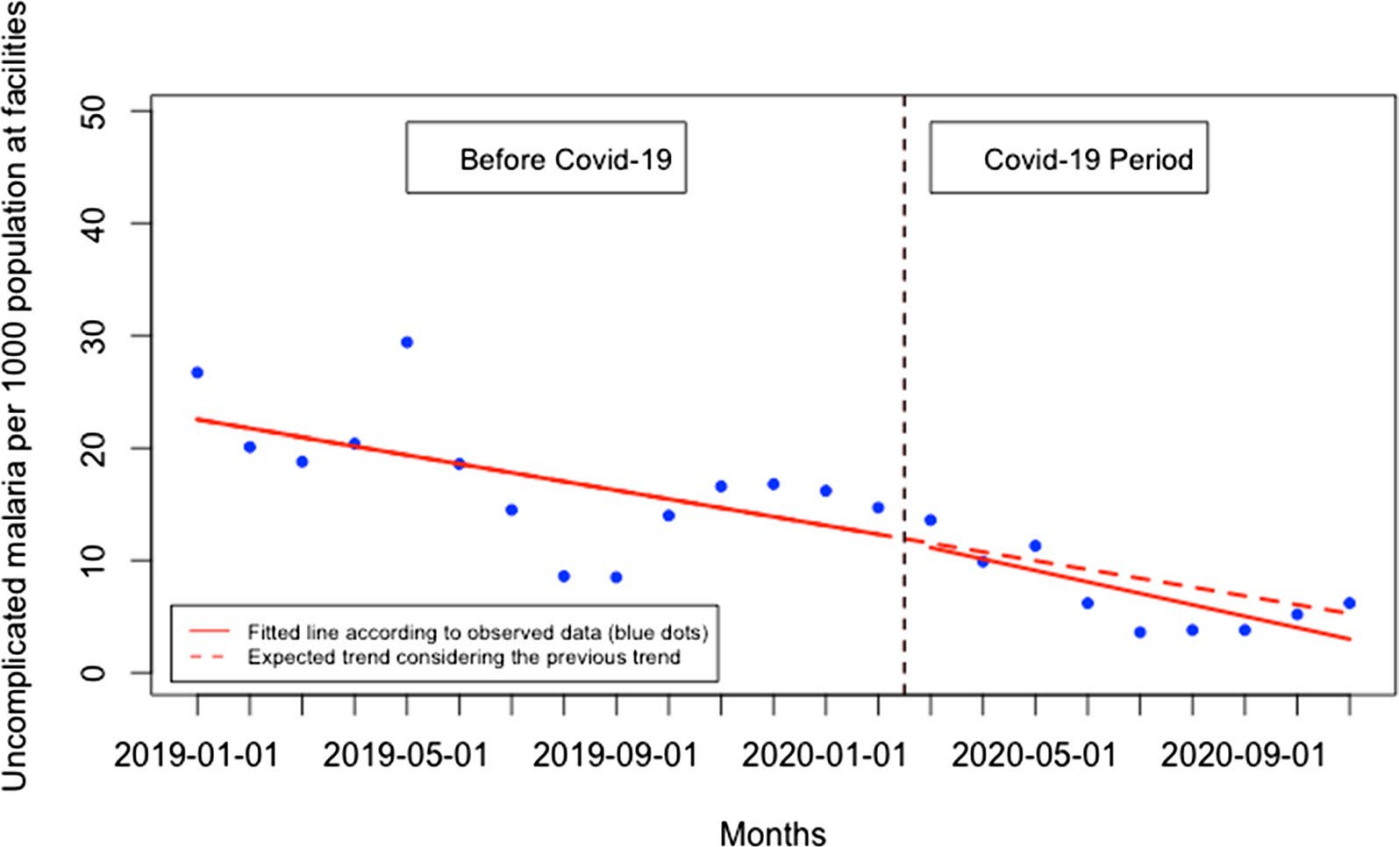
Presentation rate for uncomplicated malaria (per 1000 population) at the health facility level

**Fig. 11:**
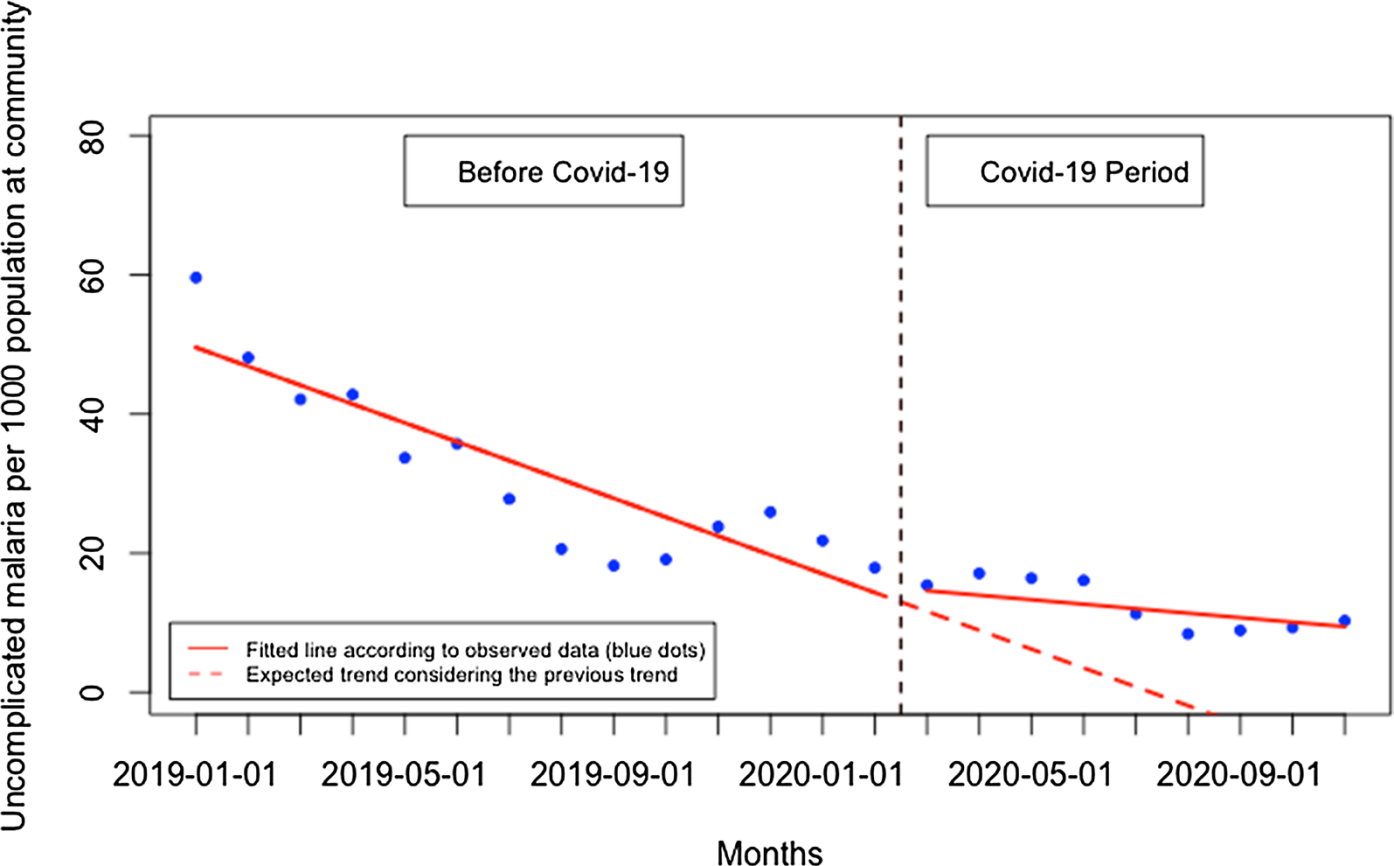
Presentation rate for uncomplicated malaria (per 1000 population) at the community level

**Fig. 12:**
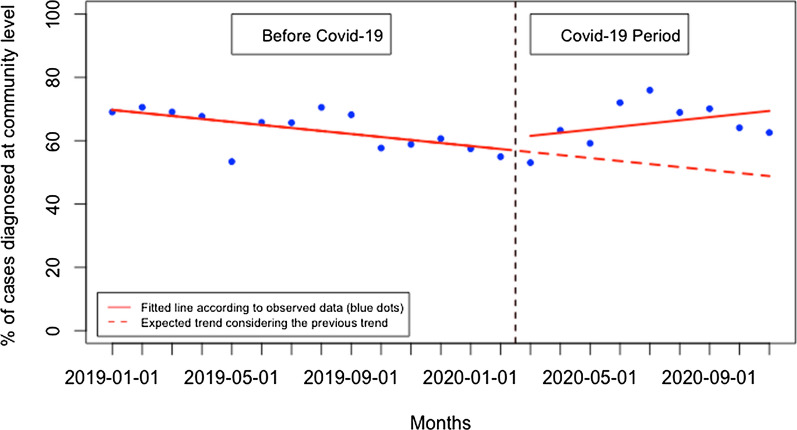
Proportion of malaria cases diagnosed at the community level

There was no significant immediate change for presentation rate for uncomplicated malaria when Covid-19 was first reported in Rwanda and there was no significant monthly change during the Covid-19 period (p > 0.05) (Fig. 9). When disaggregated, at the health facility level (Fig. 10), the presentation rate for uncomplicated malaria did not significantly change immediately when Covid-19 was first reported in Rwanda and there was no significant monthly change during the Covid-19 period (p > 0.05). At the community level (Fig. 11), the presentation rate for uncomplicated malaria did not significantly change immediately when Covid-19 was first reported in Rwanda, but there was a significant monthly increase during the Covid-19 period of 2 cases per 1000 population (95% CI 0.28, 3.84, p: 0.034). Furthermore, during the Covid-19, there was a significant monthly increase in the proportion of malaria cases diagnosed at the community level of 1.93% (95% CI 0.20, 3.66, p: 0.030) (Fig. 12).

**Fig. 13:**
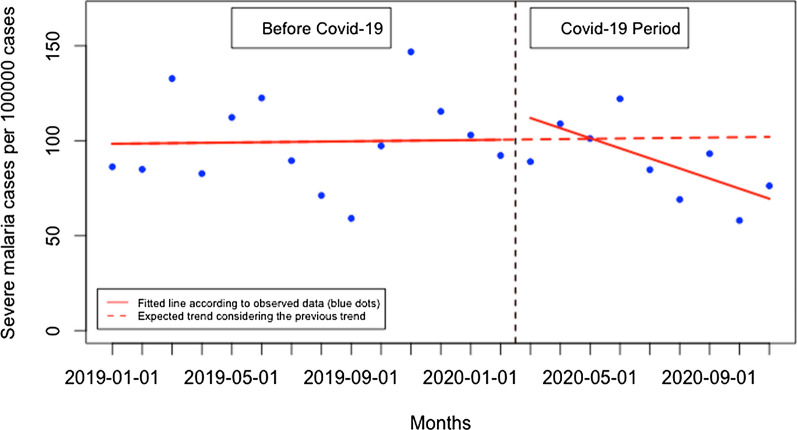
Presentation rate for severe malaria (per 100,000 malaria cases)

**Fig. 14:**
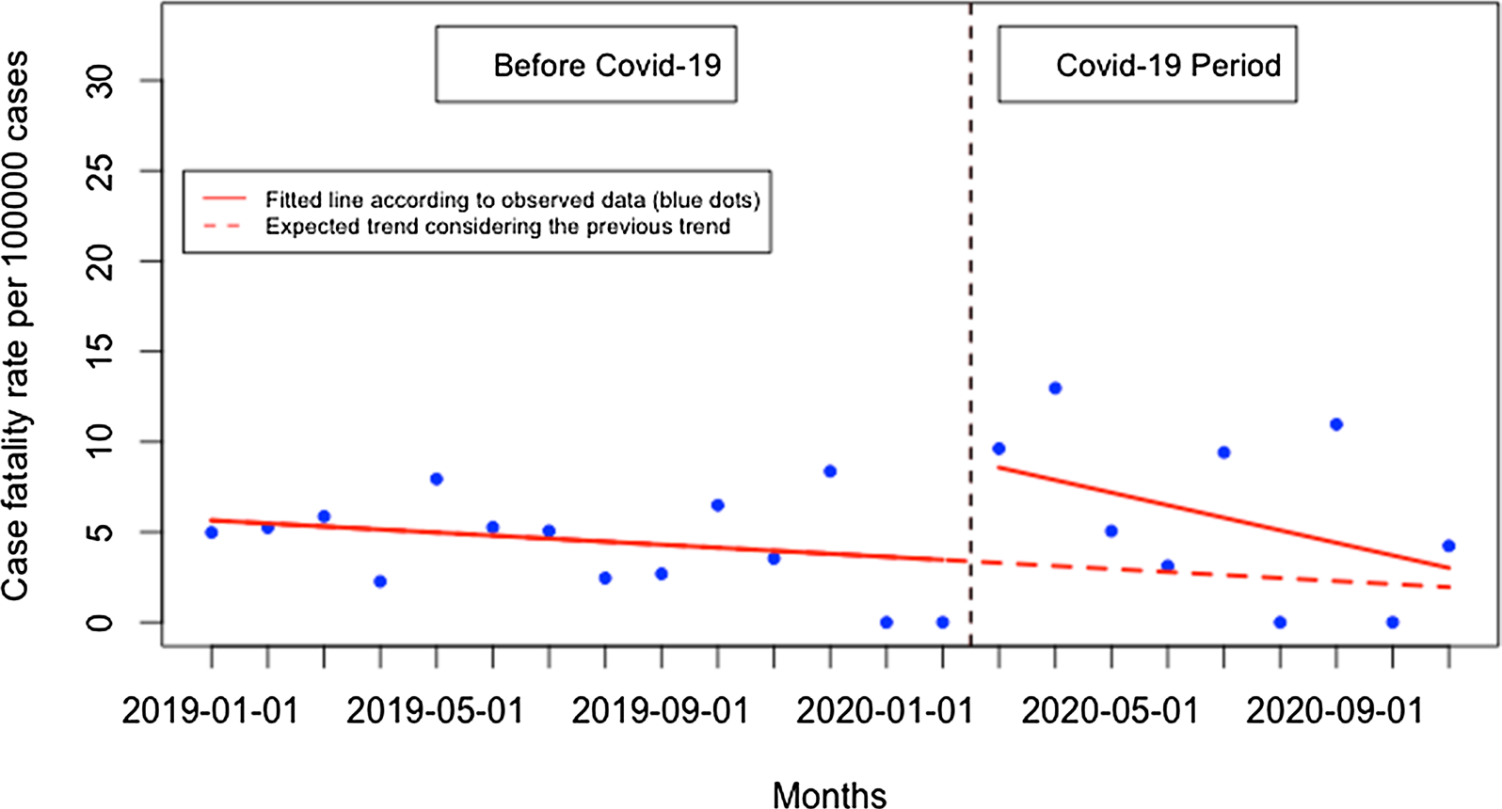
Case fatality rate per 100,000 malaria cases

The proportion of severe malaria did not significantly change when Covid-19 was first reported in Rwanda (16.74. per 100,000 malaria cases, 95% CI [− 4.68, 38.16, p: 0.142) but during the Covid-19 period, there was a significant monthly decrease in the proportion of severe malaria of 5.47 per 100,000 malaria cases (95% CI − 8.62, − 2.32, p: 0.003) (Fig. 13). Although the trend in the number of malaria deaths remained constant over time during the Covid-19 period compared to pre-Covid-19 period, there were significant changes in the malaria fatality rate. There was an increase of 5.8 deaths per 100,000 malaria cases immediately when Covid-19 case was reported in Rwanda (95% CI 3.62, 7.98, p: < 0.001) and that was followed by a significant monthly reduction of 0.52 deaths per 100,000 cases during the Covid-19 period (95% CI -0.83, -0.21, p:0.003) (Fig. 14). Detailed analysis showed that from March to November 2019, there were 28 deaths in total while there were 17 deaths during the same period in 2020. All time-series analysis results are summarized in Table 1.

**Table 1:** Impact of Covid-19 on malaria services

Malaria Indicators		Immediate change			Monthly change		
		Estimate	95% CI	P-value	Estimate	95% CI	P-value
Malaria testing	Testing (per 1000 population) the health facility level	− 5.87	[− 17.62, 5.87]	0.308	− 4.32	[− 6.25, − 2.40]	< 0.001*
	Testing (per 1000 population) at the community level	0.80	[− 9.89, 11.50]	0.884	2.38	[0.34, 4.41]	0.033*
	Testing (per 1000 population)—community and health facilities combined	− 0.43	[− 18.29, 17.42]	0.962	− 1.28	[− 5.76, 3.20]	0.582
	% of suspected malaria cases that received a parasitological test at the community level	4.75	[− 4.20, 13.70]	0.280	2.96	[1.49, 4.42]	< 0.001*
Uncomplicated malaria	Uncomplicated malaria cases (per 1000 population)	− 0.79	[− 14.55, 12.96]	0.911	2.69	[− 1.13, 6.51]	0.183
	Uncomplicated malaria cases (per 1000 population) at the health facility level	− 0.16	[− 8.02, 7.69]	0.964	− 0.23	[− 1.52, 1.05]	0.707
	Uncomplicated malaria cases (per 1000 population) at the community level	0.90	[− 7.87, 9.69]	0.841	2.06	[0.28, 3.84]	0.034*
	% of malaria cases diagnosed at the community level	3.15	[− 7.41, 13.73]	0.539	1.93	[0.20, 3.66]	0.030*
Malaria test positivity	Overall positivity rate (%)	− 1.23	[− 11.18, 8.71]	0.810	3.05	[1.22, 4.88]	0.004*
	Positivity rate (%) at the health facility level	1.93	[− 13.89, 17.77]	0.813	4.75	[1.36, 8.15]	0.012*
	Positivity rate (%) at the community level	6.72	[1.13, 12.30]	0.020*	− 0.51	[− 1.42, 0.40]	0.253
Severe malaria and deaths	Severe malaria (per 100,000 malaria cases)	16.74	[− 4.68, 38.16]	0.142	− 5.47	[− 8.62, − 2.32]	0.003*
	Case fatality rate (per 100,000 malaria cases)	5.80	[3.62, 7.98]	< 0.001*	− 0.52	[− 0.83, − 0.21]	0.003*

^*^p < 0.05—statistically significant

### Results from the qualitative analysis

The main themes were related to malaria service delivery during the Covid-19 period with more emphasis on lockdown period, perspectives about malaria service access and utilization by the community members, integration of Covid-19 messages and malaria-related messages, as well as the effect Covid-19 on the implementation of malaria core interventions.

### Malaria service delivery

CHWs and HCPs expressed that they continued to provide malaria services during the Covid-19 lockdown. However, some respondents mentioned that they were worried about how treating patients was exposing them and their families to an increased risk of contracting Covid-19. A prominent factor that caused fear among them was that the symptoms of malaria (fever, fatigue, body aches) were like Covid-19 symptoms. Additionally, most CHWs noted shortages of PPE and other basic materials early in the pandemic. As a result, few CHWs also mentioned that they refused to receive patients when the government first implemented lockdown measures.

“Our first worry was because we, community health workers, encounter many people. Our wish… if they could also give us personal protective clothing, the protective equipment would help us… our worries would reduce because we would also have access to protection.” [Community health worker, Kayonza district].

Many CHWs mentioned that the provision of masks and the designation of a team leader to oversee the re-stocking of commodities from the health centers have helped to continue providing malaria services.

“In March when this [pandemic] had just started, it disturbed us a lot, even in April. We even felt like giving up treating [patients]. But, our supervisor and the head of the health center continued to reassure us, saying that we should continue to work. Then we got lucky… at that time people hadn’t yet heard about handwashing and wearing face masks. But they [health center] called us and gave us face masks and we were able to protect ourselves. We even got the step and wash [portable hand washing stations], and that’s how we continued to work without any problems. We continued to receive patients.” [Community health worker, Rwamagana].

Some nurses and health centre staff expressed challenges at the beginning of lockdown in finding means of transportation to go to work, leading to delays in service delivery as some providers would show up late for work or would simply fail to show up. As time progressed, there was an adaptation of service delivery despite the lockdown, which improved access to the services. Many HCPs described that delivering services was later facilitated by availing designated transportation used to go and return from work, as well as designating vehicles or motorbikes to transport sick patients not able to reach the health facility. Moreover, many respondents, especially community members, reiterated that factors such as local leaders' support, improved transportation, and support from family members and friends helped to facilitate access to malaria services during the lockdown and even after.

“Because vehicles like motorbikes, cars that people use to move were not working [it was hard to get services]. But, because the police [station] is close… we live in a small rural community, exchanging information to ask them to give someone permission to let them move was easy. Even the village leader would provide them [patients] with a note and the cell office would sign it, making it easier [for them to move]. The community worker would also call [asking leaders for permission], and this would make it easy [to go seek services].” [Community member, Rwamagana].

“For us in our area, we live far from the main road. That means that our workers [nurses] had no way of getting here and during that time [lockdown], no motorbikes or vehicles were working. It became necessary that most of them take their leave days … Later, the health center had to use their motorbike to bring them to work. [Because of that], some of them used to get to work at 10, 11 am. Even, some would not come to work because they had no transport.” [Nurse, Gasabo district].

“[Getting patients] was difficult for us until we worked with the health center [to find transportation for them], and we would go get patients from their homes. Or, for patients who needed to go back home, we would put them in an ambulance that was going in or near the area they live in.” [Nurse, Kayonza district].

### Malaria service utilization

Although a few of the participants mentioned that there was no change in malaria service utilization during the lockdown, many of the respondents stated that the number of patients seeking malaria services decreased in health facilities. Reasons given for this decrease included lack of transportation, shortage of malaria commodities, fear of contracting Covid-19, and financial barriers. On the other hand, other respondents also appreciated that Covid-19 lockdown measures were implemented after houses had been sprayed with insecticides and the communities had received mosquito nets, which might have contributed to low numbers of malaria patients.

“The problem [to accessing malaria services] happened during the lockdown. You would have a sick family member but had no way of taking them to the health facility, so their illness would become severe. No cars were working, we had no other help.” [Community member, – Gasabo district].

“The number of patients during lockdown decreased. But the reason for this was because we sprayed mosquito repellents [insecticide] in 2019. That’s why we had fewer cases of malaria; we did not have many cases!” [Community health worker – Rwamagana district].

Many respondents recommended that to increase access to malaria services during the lockdown, there should be efforts to avail transportation for patients, to ensure no stockouts of malaria commodities in the community, and to involve local government representatives in campaigns to encourage community members to seek health services when feeling unwell. For many respondents, improving and strengthening the capacity of CHWs to receive patients during lockdown was believed to be the best solution to make malaria services accessible in various communities.

“If it were ever necessary for the lockdown to be reinstated, it would be good to strengthen the capacity of CHWs [to receive malaria patients] because they are closer to community members. They have the medication, the test kits, the treatment registers… undeniably, they would be able to deliver good services. If we were to be put into lockdown again, CHWs would continue to work. We have to increase the capacity of CHWs.” [Nurse-, Kayonza district].

“I think there should be a way for people to get to the health facility; maybe there should be vehicles to transport them. Let’s say, if it is a health center, it should provide an ambulance and say people who are sick in this sector can use this car to get to the hospital when other vehicles have stopped working.” [Community member, Gasabo district].

### Covid-19 and other malaria-related messages

Overall, most respondents appreciated how the government was communicating Covid-19 related information. However, they also expressed that the Covid-19 pandemic reduced the amount of information they were receiving on other diseases such as malaria and HIV. Many recommended that the government provide health-related information covering all diseases and not Covid-19 only.

“The information we get on other diseases has decreased because people have been more focused on Covid-19, that’s all they think about.” [Community member, Kayonza district]

“As things stand now, radios and televisions only focus on Covid-19 messages … I think the government should put efforts into giving [people] more messages on malaria prevention and other diseases. They should disseminate these messages on radio and television so that it’s not just Covid-19 [information] because it has distracted people.” [Community member, Gasabo district].

### Delay in implementation of some core malaria interventions

Interviews with the central level malaria programme staff showed that most of the field activities were put on hold during the Covid-19 period. For example, IRS was planned in 13 districts. However, only 10 districts were successfully sprayed by March 2020. The remaining three districts, which include the two study districts (Rwamagana, Kayonza) were sprayed with a delay of two months due to Covid-19 lockdown. Additionally, there was also a delay in Long-lasting insecticidal nets (LLINs) procurement, inspection, and distribution. Only 13 out of 30 districts were covered with LLINs by March 2020 while the remaining 17 were covered with delays extending to November 2020 due to Covid-19. Monthly reporting from the facilities to the central level did not face any challenges during the Covid-19 period. However, because in-person meetings and field activities were put on hold, there was no field data validation or feedback meetings during the Covid-19 period. These were conducted later when the government started to ease Covid-19 restrictions.

## Discussion

This study aimed to assess the impact of Covid-19 on malaria services in three districts of Rwanda. Overall, the study showed that Covid-19 mitigation measures reduced the rate of outpatient consultations in health facilities and increased consultations in the community. However, there was no impact on the uncomplicated and complicated malaria rates both immediately and over time. Nevertheless, Covid-19 mitigation measures limited the availability of transportation options for the community to seek care and delayed the implementation of some key malaria interventions. The focus on Covid-19-related communication also reduced the amount of health information for other diseases provided to community members.

This study found that Covid-19 impacted malaria services delivery. Any health crisis like the Covid-19 pandemic affects directly or indirectly the delivery of essential health services beyond malaria services delivery. For example, during the Ebola pandemic in West Africa, studies estimated a 50% reduction in access to healthcare services, a resulting increase (between 45 to 140%) in untreated cases of malaria, and more than 10,000 excess deaths added attributable to malaria [32–34]. The Ebola pandemic also affected maternal and child health services with a reduction in health facility deliveries, antenatal care utilization, and immunization among other services [35]. Relatedly, Covid-19 has impacted malaria services and other services. Findings from a recent systematic review suggested that Covid-19 disrupted the delivery of family planning and HIV services [36]. Specifically in Rwanda, Covid-19 impacted the utilization of maternal and child health services with a reduction in the utilization of antenatal care, deliveries in health facilities, postnatal care, and vaccination [37]. These findings emphasize that targeted interventions to mitigate health crisis impacts should be integral to maximize their impact. For example, efforts to mitigate the impact of Covid-19 on malaria should include improvement in immunization and antenatal care utilization to target the vulnerable populations with LLINs distribution and other malaria services [38].

The results of this study showed a significant decrease in outpatients’ consultations for malaria in health facilities. Similar findings of reduction in access to services due to Covid-19 were found in a study conducted in health facilities in 32 countries in Africa and Asia [39]. In this study, limited transport was expressed as the main reason for the decrease in outpatient consultations in health facilities. Unlike in the Global Fund study [39], fear of contracting Covid-19 was not expressed as a primary barrier to seeking malaria care. This difference may be because, in Rwanda, community members have high trust in health services. Moreover, there was constant communication about Covid-19 to the community even before Covid-19 was reported in Rwanda. The messages included the readiness of health facilities to prevent Covid-19 transmission. Thus, the community members were likely knowledgeable about Covid-19, which might have reduced the fear to come to the health facilities. While further explorations are needed to better understand the root causes of the decrease of presentations in health facilities, the increase in the community cases suggests that some community members who were getting care in health facilities may have shifted to CHWs during the Covid-19 period. This reiterates the importance of bringing care closer to the community and exemplifies the value of the CHWs in ensuring continuous delivery of essential health services especially during pandemics that may require lockdown. However, CHWs are already overburdened with many other health-related activities [40]. As more people may continue to use community services, the malaria programme should ensure that the programme of home-based management of malaria is well-equipped and CHWs are not overburdened by an increase in a number of patients. Strategies such as training other CHWs who were not treating malaria would help to unburden those treating malaria.

This study showed an immediate increase in the case fatality rate followed by a constant decrease during the Covid-19 period. Explanations may include how this indicator is calculated. The number of deaths remained very low but constant overtime while there has been a constant decrease in malaria cases even before Covid-19 was first reported in Rwanda. On the other hand, in Rwanda, the only source of data for confirmed malaria-related deaths is HMIS, which captures deaths that occurred at health facilities or those that happened in the community and brought to a health facility for autopsy, a service that is still limited in Rwanda. The recent WHO malaria report showed that globally, there was a 12% increase in malaria deaths in 2020 compared to 2019, and half of the deaths occurred in just six African countries. More importantly, 68% of the additional deaths were due to service interruptions during the Covid-19 pandemic [41]. Although Rwanda was not among six countries with many deaths, the disruptions of services would still impact health indicators including malaria deaths. Deaths might have happened in the community and been left undocumented. The data on community-related deaths are not available and this study could not capture community deaths. Further explorations are needed to ascertain that there were no patients that died in the community due to malaria. Moreover, the study findings also highlight a need to strengthen community death reporting such as community verbal autopsy.

Additionally, the study findings reveal that Covid-19 mitigation measures caused delays in the implementation of core malaria interventions including the IRS campaign, LLINs distribution, and delay in data validation, although the reporting rate remained high. The high reporting rate despite Covid-19 challenges may be due to the use of electronic reporting system across health facilities. Existing evidence suggests that disruption in the implementation of malaria prevention interventions could result in a significant increase in malaria cases and deaths [16, 42]. Although the study findings did not show a significant change in uncomplicated malaria rates, the study was limited to nine months and could not assess the long-term impact of delayed interventions on malaria cases. This obliges malaria programmes to timely speed up the implementation of delayed interventions. The IRS campaign and LLNs distribution should be timed to match with the malaria transmission season to mitigate the long-term consequences. Moreover, there is a need to adapt the implementation of the core interventions, such as distribution of LLINs through CHWs or other existing institutions such as schools and community-based organizations, which has been shown to improve LLINs coverage in the community, especially in hard-to-reach areas and during emergencies [15, 43, 44].

In this study, the participants expressed that efforts to provide Covid-19-related information reduced other health-related messages. Covid-19 is a pandemic that has global attention. All countries are on high alert to curb its increase. However, if all resources and attention are directed only to Covid-19, other programmes including the malaria programme would be left unsupported and thus undermine achievements made. While Covid-19 related messages are still important to address rumors and misconceptions, the study findings highlight a need to integrate Covid-19 related messages with other messages [45]. Such integration would not only promote efficient use of the available resources but also help in ensuring that community members stay knowledgeable about other health topics and continue to use health services.

This study also intended to assess the impact of Covid-19 on the supply of malaria commodities in the community. However, this analysis was not possible mainly due to the inaccessibility of the electronic Logistics Management Information System (eLMIS), a system used to report stockouts of malaria commodities from the community and facilities. There have been ongoing changes in the system, and they were not yet completed at the time of data collection to avail quality data. Additionally, the attempts to extract data from the paper-based reports in health facilities failed because some of the reports were unreadable, and others were not available. While further analyses of the stock status of malaria commodities are recommended to better understand the impact of Covid-19 on the supply of malaria commodities, especially at the community level, in the qualitative study, participants did not report major challenges in accessing malaria commodities. Moreover, the increase in patients tested in the community during the Covid-19 period can indirectly suggest that there were no major challenges with commodity supply at the community level. Considering these challenges observed, it is worth noting that the programme should speed up the process of optimizing and scaling up the Logistics Management Information System and possibly consider using other digital forms in managing commodities and other community health services to tackle archiving challenges.

The results of this study should be interpreted considering its limitations. The time-series analysis did not have a control group and used retrospective data. Thus, the study could not control all possible confounding factors other than the time effect. Moreover, being a cross-sectional study within a limited period, it could not assess the long-term impact of delayed services on malaria cases. Furthermore, this study was only limited to three districts of the 12 malaria-endemic districts and may not be representative of the whole country. Further studies are needed to assess the situation in other geographical locations.

## Conclusion

This study demonstrated that the Covid-19 pandemic shifted patients’ consultations from health facilities to the community and that implementation of malaria core interventions was delayed. Moreover, limited transportation due to Covid-19 mitigation measures posed a challenge for the community in seeking malaria care. Widespread Covid-19 communication messages also reduced the presence and impact of other health-related messages. To reverse Covid-19’s impact on malaria service delivery and utilization, programmes should speed up and sustain the interrupted IRS campaign and LLINs distribution. Moreover, investing in CHWs to strengthen home-based malaria management, and other community and home-based care models needs to be prioritized, and the strategic integration of malaria messages into Covid-19-related communication should be prioritized. Further studies in other geographical locations and on the long-term impact of disrupted malaria interventions are needed.

## Data Availability

All relevant data are presented in the paper. The data that support the findings of this study are available from Health Concepts and Innovation Solutions and the Ministry of Health—Rwanda Biomedical Center but restrictions apply to the availability of these data, which were used under license for the current study, and so are not publicly available. Data are however available from the authors upon reasonable request and with permission of Health Concepts and Innovation Solutions and the Ministry of Health—Rwanda Biomedical Center.

## Abbreviations

%: Percentage
CHWS: Community Health Workers
CI: Confidence Intervals
HCPs: Health Care Providers
HICS: Health Concepts and Innovation Solutions
HMIS: Health Management Information System
IRS: Indoor Residual Spraying
LLINS: Long-lasting insecticidal nets
RBC: Rwanda Biomedical Center
SISCOM: Système Informatique de Santé Communautaire
UGHE: University of Global Health Equity
WHO: World Health Organization
